# Supplementary figures and images for: Transversal sero-epidemiological study of Bordetella pertussis in Tehran, Iran
Source: PLoS One. 2020 Sep 1;15(9):e0238398. doi: 10.1371/journal.pone.0238398 (PMC7462262; doi:10.1371/journal.pone.0238398)

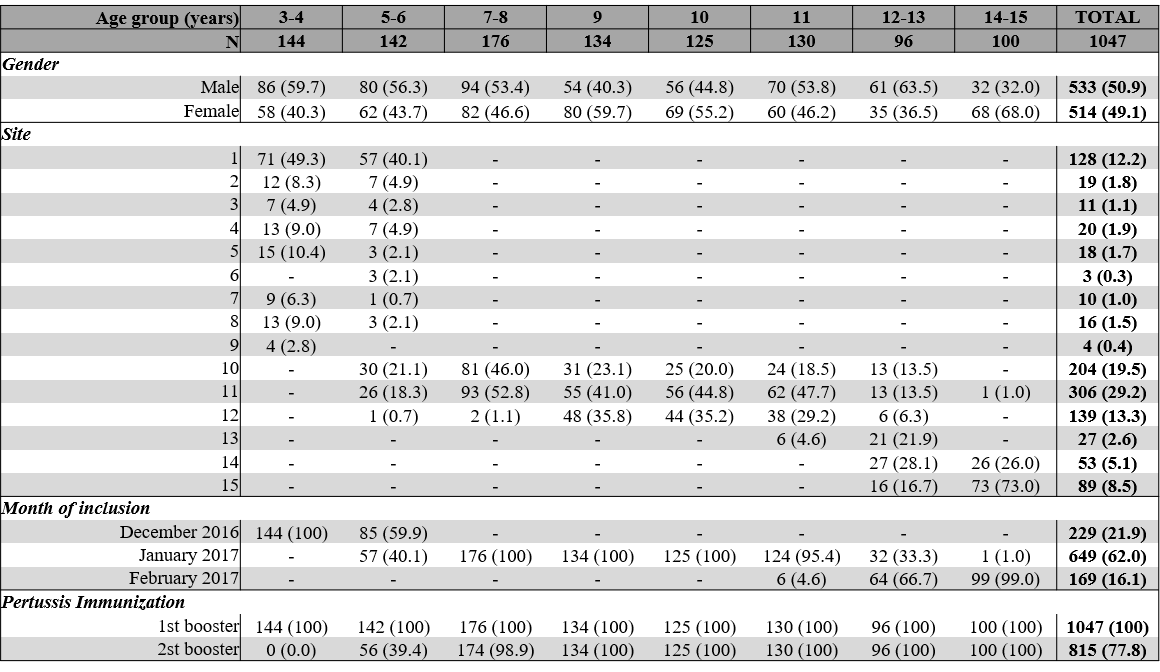

Supplement: S1 Table — Values are shown as n (%). (TIF) [file pone.0238398.s001.tif]

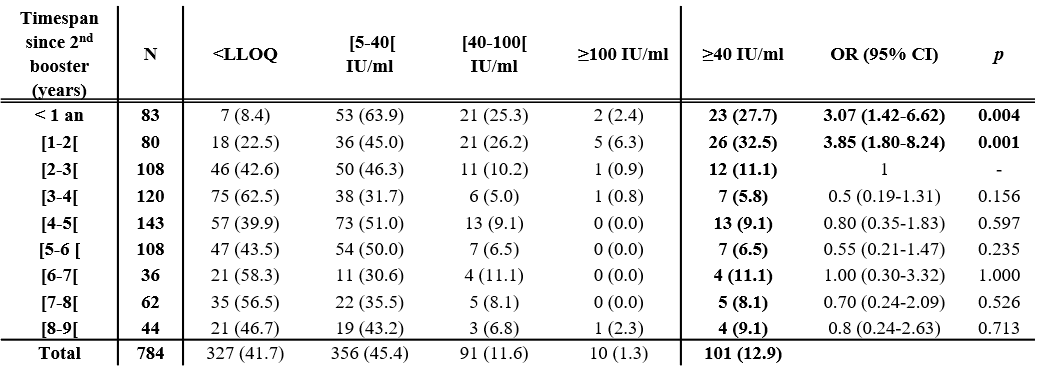

Supplement: S2 Table — Anti-PT IgG levels are shown using <LLOQ, [5–40[, [40–100 [and ≥100 IU/ml cut-offs, and the ≥40 IU/ml cut-off alone to include all anti-PT positive individuals. Anti-PT IgG titers are shown as n (%). (TIF) [file pone.0238398.s002.tif]
